# A combined blood based gene expression and plasma protein abundance signature for diagnosis of epithelial ovarian cancer - a study of the OVCAD consortium

**DOI:** 10.1186/1471-2407-13-178

**Published:** 2013-04-03

**Authors:** Dietmar Pils, Dan Tong, Gudrun Hager, Eva Obermayr, Stefanie Aust, Georg Heinze, Maria Kohl, Eva Schuster, Andrea Wolf, Jalid Sehouli, Ioana Braicu, Ignace Vergote, Toon Van Gorp, Sven Mahner, Nicole Concin, Paul Speiser, Robert Zeillinger

**Affiliations:** 1 Department of Obstetrics and Gynecology, Molecular Oncology Group, Medical University of Vienna, European Union, Vienna, Austria; 2 Ludwig Boltzmann Cluster “Translational Oncology”, General Hospital Vienna, European Union, Waehringer Guertel 18-20, Room-No.: 5.Q9.27, Vienna, A-1090, Austria; 3 Section for Clinical Biometrics, Center for Medical Statistics, Informatics and Intelligent Systems, Medical University of Vienna, European Union, Vienna, Austria; 4 Department of Gynecology, Campus Virchow Klinikum, Charite Medical University, European Union, Berlin, Germany; 5 Department of Obstetrics and Gynecology, Division of Gynecological Oncology, University Hospitals Leuven, Katholieke Universiteit Leuven, European Union, Leuven, Belgium; 6 Division of Gynaecological Oncology, Department of Obstetrics and Gynaecology, MUMC+, GROW – School for Oncology and Developmental Biology, European Union, Maastricht, The Netherlands; 7 Department of Gynecology and Gynecologic Oncology, University Medical Center Hamburg-Eppendorf, European Union, Hamburg, Germany; 8 Department of Gynecology and Obstetrics, Innsbruck Medical University, European Union, Innsbruck, Austria

**Keywords:** Peripheral blood leukocytes, Biomarker, Transcriptomics, Plasma protein, Diagnosis, Ovarian cancer

## Abstract

**Background:**

The immune system is a key player in fighting cancer. Thus, we sought to identify a molecular ‘immune response signature’ indicating the presence of epithelial ovarian cancer (EOC) and to combine this with a serum protein biomarker panel to increase the specificity and sensitivity for earlier detection of EOC.

**Methods:**

Comparing the expression of 32,000 genes in a leukocytes fraction from 44 EOC patients and 19 controls, three uncorrelated shrunken centroid models were selected, comprised of 7, 14, and 6 genes. A second selection step using RT-qPCR data and significance analysis of microarrays yielded 13 genes (AP2A1, B4GALT1, C1orf63, CCR2, CFP, DIS3, NEAT1, NOXA1, OSM, PAPOLG, PRIC285, ZNF419, and BC037918) which were finally used in 343 samples (90 healthy, six cystadenoma, eight low malignant potential tumor, 19 FIGO I/II, and 220 FIGO III/IV EOC patients). Using new 65 controls and 224 EOC patients (thereof 14 FIGO I/II) the abundances of six plasma proteins (MIF, prolactin, CA125, leptin, osteopondin, and IGF2) was determined and used in combination with the expression values from the 13 genes for diagnosis of EOC.

**Results:**

Combined diagnostic models using either each five gene expression and plasma protein abundance values or 13 gene expression and six plasma protein abundance values can discriminate controls from patients with EOC with Receiver Operator Characteristics Area Under the Curve values of 0.998 and bootstrap .632+ validated classification errors of 3.1% and 2.8%, respectively. The sensitivities were 97.8% and 95.6%, respectively, at a set specificity of 99.6%.

**Conclusions:**

The combination of gene expression and plasma protein based blood derived biomarkers in one diagnostic model increases the sensitivity and the specificity significantly. Such a diagnostic test may allow earlier diagnosis of epithelial ovarian cancer.

## Background

One of the most deadly malignant diseases in women is ovarian cancer. The high risk of dying is particularly due to late diagnosis, i. e. 67% of patients are diagnosed with advanced disease. The five-year overall survival (OS) rate is only 46% among all stages [1]. Patients with stage I disease have a five-year OS rate of about 90%, whereas patients with advanced disease less than 30% [2]. One reason for the low five-year OS rate is the fact that ovarian cancer presents with few, if any, specific symptoms. Therefore markers for early detection of ovarian cancer could improve OS.

Up to now no screening markers are recommended or routinely used for early detection of ovarian cancer. One of the known serum marker for ovarian cancer is CA-125, described for the first time in 1981 as a murine monoclonal antibody (OC125) reacting against ovarian cancer cell lines and cryopreserved ovarian cancer tissues but not against benign tissues or other carcinomas [3]. CA-125 is a coelomic epithelial antigen produced by mesothelial cells in the peritoneum, pleural cavity and pericardium and in several other epithelia such as the gastrointestinal tract, respiratory tract, and genital tract. Serum CA-125 levels are measurably increased in about 80% of patients with ovarian cancer. An increase is measured to a lesser extent in patients with early stages, resulting in a sensitivity of CA-125 screening of lower than 60% in early stages [4]. Serum concentrations can be elevated by a number of common benign gynecologic conditions, including endometriosis and leiomyomas, as well as by non-gynecologic pathologies such as congestive heart failure and liver cirrhosis. In general, serum concentrations of CA-125 are higher in premenopausal women, compared to postmenopausal women. These facts all together results in an impaired sensitivity and specificity for CA-125 [5]. Nevertheless, there are numerous papers dealing with CA-125 as marker for early detection, diagnosis, response prediction and monitoring, disease recurrence, and for distinguishing malignant from benign pelvic tumors [6].

To increase the sensitivity and specificity of CA-125, this single marker could be expanded to a marker panel. Including other serum markers and building a statistical model, this might result in a more sensitive and specific signature for detection of EOC.

In 2004 Zhang et al. published a four marker panel comprised of CA-125 and three by mass spectroscopy (SELDI) newly identified serum protein peaks, identified as apolipoprotein A1 (down-regulated in malignant tumors), a truncated form of transthyretin (down-regulated), and a cleaved fragment of inter-α-trypsin inhibitor heavy chain H4 (up-regulated) [7]. A multivariate model combining the three biomarkers and CA-125 reached a sensitivity of 74% by a fixed specificity of 97% for detection of early stage EOC. This set of biomarkers was amended by four additional serum protein peaks leading to a commercialized FDA cleared blood test for assessment of the likelihood that an ovarian mass is malignant, called OVA1™ (Quest Diagnostics, Madison, NJ, USA). Recently, in a prospective study, the effectiveness of the OVA1™ test was compared to the malignancy-assessment by physicians. The multivariate index assay demonstrated higher sensitivity and lower specificity compared to the physician assessment together with the CA-125 serum levels [8,9].

Mor et al. described in 2005 four new serum markers, namely Leptin, Prolactin, OPN, and IGF-II, found by a rolling circle amplification (RCA) immunoassay microarray approach. In a combined predictive model including 19% early stage patients, an overall sensitivity and specificity of approx. 95% was reached [10]. Adding CA-125 and MIF to this four-marker-panel, the specificity was increased to 99.4% at a sensitivity of 95.3%. With this marker panel, 11.1% of stage I and II samples (4 of 36) were misclassified [11].

Recently, Yurkovetsky et al. described a four serum marker panel, namely HE4, CEA, VCAM-1, and CA-125, for early detection of ovarian cancer. A model derived from these four serum markers provided a diagnostic power of 86% sensitivity for early stage, and 93% sensitivity for late stage ovarian cancer at a specificity of 98% [12].

Another approach to find prognostic markers for early detection of ovarian cancer is to use peripheral blood cells instead of serum. In 2005 a set of 37 genes was identified whose expression in peripheral blood cells could detect a malignancy in at least 82% of breast cancer patients [13]. Very recently, a set of 738 genes was identified discriminating breast cancer patients from controls with an estimated prediction accuracy of 79.5% (80.6% sensitivity and 78.3% specificity) [14].

The aim of this study was to investigate if combining gene-expression patterns with a serum protein panel results in a more sensitive and more specific signature for the detection of EOC. Primarily, we isolated a leukocytes fraction from epithelial ovarian cancer (EOC) patients, patients with non-malignant gynecological diseases and healthy blood donors (controls). A whole genome transcriptomics approach (Applied Biosystems Human Genome Survey microarrays V2.0) was used to identify gene expression patterns discriminating between ovarian cancer patients and healthy controls or patients with non-malignant diseases. In the second place we determined a six-protein panel [11] from the plasma samples. Taken together predictive models were built from a large cohort of patients and controls using either RT-qPCR derived expression values or protein abundance values alone or in combination. Validation was performed by means of the bootstrap .632+ cross-validation method.

## Methods

### Patients and controls

In total, blood from 239 epithelial ovarian cancer (EOC) patients (19 FIGO I/II and 220 FIGO III/IV) and 169 controls (120 healthy blood donors and 49 patients with benign ovarian tumors (cystadenomas) or low malignant potential (LMP) tumors) were enrolled in this retrospective study (Table 1). Controls, including healthy blood donors and patients with benign gynecologic diseases, were collected chronologically at the Medical University of Vienna, Austria, during one year, thus representing a cross-section of the population at risk. All blood samples from epithelial ovarian cancer patients were collected in the course of the EU-project OVCAD (Ovarian Cancer - Diagnosing a Silent Killer) within two days prior to surgery (Charité, Berlin Medical University, Germany n = 86, University Medical Center Hamburg-Eppendorf, Germany n = 43, Medical University of Innsbruck, Austria n = 11, Katholieke Universiteit Leuven, Belgium n = 52, Medical University of Vienna, Austria n = 47). Informed consent for the scientific use of biological material was obtained from all patients and blood donors in accordance with the requirements of the local ethics committees of the involved institutions. Clinicopathologic parameters were assessed by the specialized pathologists at each participating university hospital according to reviewed OVCAD criteria.

**Table 1:** Overall statistics for EOC patients, patients with benign or low malignant potential (LMP) tumors, and healthy persons and patients with benign diseases as controls (A), clinicopathologic characteristics of FIGO I/II and FIGO III/IV patients (B) and diagnosis of patients with benign diseases (C)

**A)**					
**Cohort 1**	**Typ**	**Number**	**FIGO**	**Age ± SD [years]**	**Range [years]**
Controls	Healthy	90	n. a.	46.7 ± 16.8	19 - 83
	Cystadenoma	6	n. a.	57.3 ± 8.5	45 - 66
	LMP	8	n. a.	60.0 ± 18.6	32 - 92
Malignant disease	Ovarian cancer	19	FIGO I-II	55.5 ± 16.7	15 - 85
		220	FIGO III-IV	58.6 ± 11.8	18 - 83
**Cohort 2**					
Controls	Healthy	30	n. a.		
	Benign gynecological diseases	35	n. a.	47.3 ± 13.2	25 - 74
Malignant disease (overlapping with cohort 1)	Ovarian Cancer	14	FIGO I-II		
		210	FIGO III-IV		
**B)**					
**FIGO I-II patients**		19			
Histology					
Serous		14			
Endometrioid		2			
Mucinous		1			
Undifferentiated		2			
FIGO					
Ia		2			
Ic		7			
IIa		4			
IIb		2			
IIc		4			
Grade (1 missing)					
1		4			
2		6			
3		8			
**FIGO III-IV patients**		220			
Histology (1 missing)					
Serous		194			
Endometrioid		4			
Mucinous		3			
Undifferentiated		6			
Mixed epithelial		12			
FIGO (3 missing)					
IIIa		4			
IIIb		7			
IIIc		166			
IV		40			
Grade (4 missing)					
1		8			
2		51			
3		157			
**C)**					
**Benign diseases**		35			
Cystadenoma (mucinous)		9			
Endometriosis		5			
Ovarian fibroma		2			
Uterine myoma		9			
Miscellaneous (two with inflammatory conditions)		10			

### Isolation of the leukocytes fraction and total RNA preparation

A leukocytes fraction depleted from epithelial cells was isolated from EDTA-blood by a density gradient centrifugation protocol, largely according to Brandt and Griwatz [15]. Total RNA was isolated using the RNeasy Mini kit (QIAGEN, Venlo, Netherlands) and quality-checked with the Agilent 2100 Bioanalyzer (Agilent Technologies, Santa Clara, Ca, USA). The RNA-quantity was measured spectrophotometrically.

### Microarray analysis and pre-selection

Whole genome expression analysis was performed on single channel Applied Biosystems Human Genome Survey microarrays V2.0 (Applied Biosystems, Foster City, Ca, USA) containing 32,878 probes representing 29,098 genes. Two μg total RNA from 44 ovarian cancer patients and 19 age-matched controls (13 completely healthy controls and 6 patients with benign ovarian cysts (mean 60.8 ± 13.7 years and 61.7 ± 12.9 years, respectively) were labeled with the NanoAmp RT-IVT Labeling Kit and hybridized to the microarrays for 16 hours at 55°C. After washing and visualization of bound digoxigenin-labeled cRNAs with the Chemiluminescence Detection Kit according to the manufacturer’s instructions (Applied Biosystems), images were read with the 1700 Chemiluminescent Microarray Analyzer (Applied Biosystems). Raw expression data, signal-to-noise ratios and quality-flags delivered from the Applied Biosystems Expression System software were further processed using Bioconductor's ABarray package (http://www.bioconductor.org). In brief, raw expression values were log_2_ transformed and measurements with quality indicator flag values greater than 5000 were set missing. For inter-array comparability, data were quantile-normalized and missing values imputed with 10-nearest neighbors imputation. Several pre-filtering steps of probes were performed. Firstly, 13,520 probeIDs which exhibited a signal-to-noise ratio less than 2 in at least 50% of the two pooled groups (patients with malignant disease and non-malignant controls) were excluded (19,358 probeIDs were remaining). Secondly, 10,125 probeIDs assumed to be potentially affected by batch-effects were excluded, resulting in remaining 9,233 probeIDs. Finally, 205 probeIDs with fold-changes > 3 between both groups were selected. Three further genes were eliminated due to non-available TaqMan® Assay-on-Demand probes and primer sets (Applied Biosystems). From the remaining 202 probeIDs three consecutive predictive models were built using the uncorrelated shrunken centroids (USC) [16] approach with default parameters, implemented in the MultiExperiment Viewer (MeV) [17]. This methods selects uncorrelated genes which best discriminate the two groups in internal cross-validation. Since the method picks only one gene from a group of several highly correlated genes, and this selection may be arbitrarily affected by small-sample variation, we repeated the method twice each time excluding the genes found in the previous step. This iterative approach leads to a richer set of candidate genes for further analyses. Microarray data are accessible on the Gene Expression Omnibus (GEO) under GEO accession: GSE31682.

### Evaluation of microarray results by RT-qPCR

The microarray gene expression measurements of the selected genes were validated by real time RT-qPCR. cDNA was synthesized from 1 μg total RNA using the M-MLV reverse transcriptase (Promega, Madison, WI, USA) and a random nonamer primer. For normalization three stably expressed genes were selected from all 63 microarrays and all genes with signal-to-noise ratios greater than 3 in all samples (8,318 probeIDs): RPL21 (Ribosomal protein L21, Assay-on-Demand TaqMan® probe: Hs03003806_g1), RPL9 (Ribosomal protein L9, Hs01552541_g1), and SH3BGRL3 (SH3 domain-binding glutamic acid-rich-like protein 3, Hs00606773_g1), with coefficients of variation (CV) of 0.014, 0.012, and 0.014, respectively. The geometric mean of the RT-qPCR values of these three normalizers was calculated for each sample and this normalizing sample-specific constant was subtracted from each measurement of sample to obtain normalized (delta-CT) values. Delta-CT values were finally multiplied by −1 to be interpretable as log_2_-expression values.

### Determination of the six-protein panel

The abundances of the six proteins (MIF, prolactin, CA125, leptin, osteopondin, and IGF2) from the cancer biomarker panel [11] were determined from the plasma samples according to the MILLIPLEX MAP Kit – Cancer Biomarker Panel (Millipore, Billerica, MA, USA) using the Luminex technology on the Bio-Plex 200 System (Bio-Rad Laboratories, Hercules, Ca, USA).

### Statistical analysis and model building

Differences in mean age between the five clinically defined groups (Table 1) were assessed by analysis of variance (ANOVA), followed by Tukey’s post hoc tests. Significant up- or down-regulation of the expression of the 13 genes (AP2A1, B4GALT1, C1orf63, CCR2, CFP, DIS3, NEAT1, NOXA1, OSM, PAPOLG, PRIC285, ZNF419, and BC037918) and the 6 proteins between healthy controls and patients with malignant disease (extra for FIGO I/II and FIGO III/IV patients) was assessed by t tests followed by correction for multiple testing by the Holm–Bonferroni method.

For selection the log_2_ expression values from 20 genes were compared between samples from healthy patients and patients with malignant tumors by the significance analysis of microarrays (SAM) procedure, employing the t statistic and using R's samr package [18]. 13 Genes with q-values less than 0.15 were finally selected for model building with data from cohort 1. To this end the expression of these genes were determined by RT-qPCR in all 239 malignant (including the 44 ovarian cancer patients from the microarray experiment), 90 healthy (including 13 of the 19 controls from the microarray experiment), and 14 low-malignant potential or benign samples. Gene expression values were normalized as described above, and an *L*_1_ penalized logistic regression model, also known as LASSO, which retained all 13 genes was estimated to obtain a model discriminating between the healthy and diseased groups [19].

**Figure 1:**
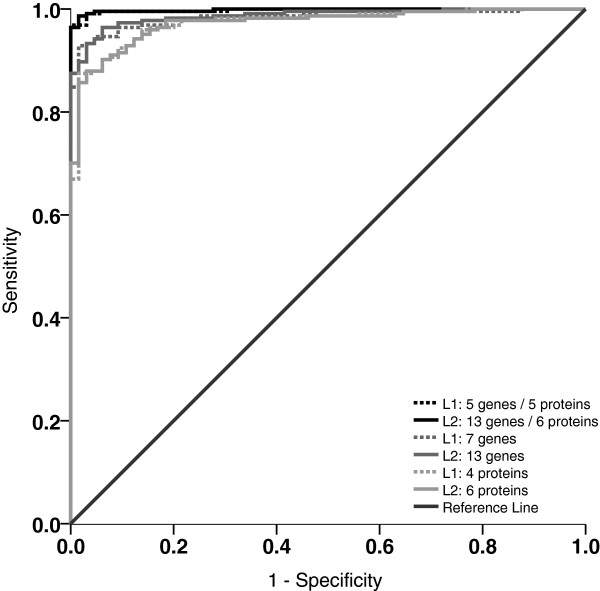
**Area under the receiver operating characteristic (ROC) curves (AUCs) for all six models built from blood based expression values and/or plasma based protein abundances as derived from cohort 2 (for key metrics see Figure**2**and Table**6**).**

Unfortunately, the plasma samples from the original 90 healthy controls were not available and therefore a further cohort of 65 controls (30 healthy blood donors and 35 patients with benign gynecological diseases) was enrolled in the study (cohort 2). The expressions of the 13 genes and the abundances of the six proteins were determined as described above. Using these two groups, one comprised of 224 EOC patients (for the remaining 15 EOC samples, no plasma samples were available) and one comprised of 65 controls (cohort 2), models using either gene expression values or protein abundance values alone or both in combination were built by means of L1 and L2 penalized logistic regressions, also known as LASSO and ridge regression, respectively (*cf.* Figure 1 for ROCs). Both models impose a penalty on the regression coefficients such that the sum of their absolute values (L1) or the sum of their squared values (L2) does not exceed a threshold value λ. The optimal value of the tuning parameter λ is found by maximizing the leave-one-out cross-validated likelihood. While L1 penalized models may set some regression coefficients exactly to zero, thus selecting a subset of the variables as predictors, L2 models always include all variables. The glmpath R package was used for computing the L1 and L2 models. To assess the differences of the obtained discriminatory models, likelihood ratio tests were performed.

### Bootstrap validation

The misclassification error rate and the cross-validated receiver operating characteristic curve were estimated using the bootstrap .632+ cross-validation procedure [20].

## Results

### Gene expression based biomarkers

**Figure 2:**
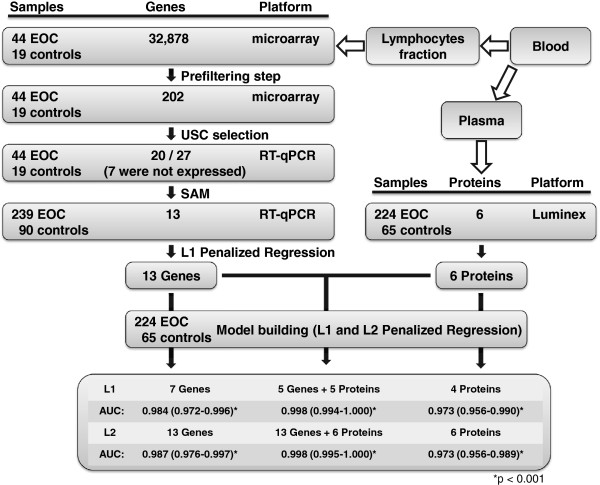
**Outline of the pre-selection, the selection, the model building, and the validation procedure.** (EOC, epithelial ovarian cancer; USC, uncorrelated shrunken centroids; SAM, significance analysis of microarrays; LASSO, L1 penalized logistic regression model; AUC, area under the receiver operating characteristic (ROC) curve; LMP, low malignant potential; n. s., not significant).

Figure 2 outlines the gene selection and model building procedure for the mRNA-expression based genes. Starting from 202 genes preselected as described above, three consecutive uncorrelated shrunken centroid (USC) models were built, comprised of 7, 14, and 6 genes, respectively. Expressions of these 27 genes were validated in 63 samples using RT-qPCR with corresponding Assay-on-Demand TaqMan® probes (Table 2) and a set of three stably expressed genes as normalizers, selected also from the microarray data. Seven of these 27 failed the validation step, because these genes showed no expressions in the 63 samples, indicating microarray artifacts or problems with the Assay-on-Demand TaqMan® probes (Table 2). A further selection step by Significance Analysis of Microarrays (SAM) selected 13 of the remaining 20 genes with q-values ≤ 0.15 (Table 2).

**Table 2:** **Gene list of the 27 genes from the three USC-models, corresponding Assay-on-Demand TaqMan® probes, SAM-results from the second selection step, and coefficients of the final L1 penalized logistic regression model**

**Genes**		**Evaluation**		**SAM**	**L1 model (13 genes)**
**ProbeID**	**Gene symbol**	**TaqMan**^**® **^**probe**	**RT-qPCR**	**q-value (**≤ **0.15)**	**Coefficient**
**USC model 1**					
119290	CFP	Hs00175252_m1	yes	**0.13**	1.241
182018	NOXA1	Hs01017917_m1	yes	**0.09**	−0.888
184360	RETNLB	Hs00395669_m1	no		
212552	ZNF546	Hs00418908_m1	no		
228089	NEAT1	Hs01008264_s1	yes	**0.01**	2.075
713562	N/A (BC037918)	Hs00860048_g1	yes	**0.01**	0.035
10546171	N/A	Hs01036865_m1	no		
**USC model 2**					
105700	AMZ1	Hs00401010_m1	no		
105743	DIS3	Hs00209014_m1	yes	**0.10**	1.177
109227	ZNF419	Hs00226724_m1	yes	**0.08**	0.145
110071	CCR2	Hs00356601_m1	yes	**0.01**	0.376
110496	DYSF	Hs00243339_m1	yes	0.49	not used
118384	HGS	Hs00610371_m1	yes	0.39	not used
136788	ALX4	Hs00222494_m1	no		
142487	B4GALT1	Hs00155245_m1	yes	**0.11**	−0.642
160314	DBNL	Hs00429482_m1	yes	0.50	not used
161219	MPP1	Hs00609971_m1	yes	0.41	not used
161567	PAPOLG	Hs00224661_m1	yes	**0.01**	−0.454
162222	PRIC285	Hs00375688_m1	yes	**0.09**	−1.794
223870	CCL3L1	Hs00824185_s1	yes	0.32	not used
224628	ANKHD1	Hs00226589_m1	yes	0.24	not used
**USC model 3**			no		
115368	AP2A1	Hs00367123_m1	yes	**0.15**	−0.199
157342	C1orf63	Hs00220428_m1	yes	**<0.01**	−0.230
177183	RMI1	Hs00227878_m1	yes	0.37	not used
204670	GRM1	Hs00168250_m1	no		
205406	OSM	Hs00171165_m1	yes	**0.01**	−1.105
220229	ASGR1	Hs00155881_m1	no		
					Intercept: 6.320

Normalized RT-qPCR expression values of these 13 genes were determined from all 343 samples of cohort 1. Regulation levels for each FIGO group, FIGO I/II and FIGO III/IV, are shown in Table 3A. Five genes were significantly down-regulated in the leukocytes fraction of FIGO I/II and FIGO III/IV EOC patients compared to 90 healthy blood donors, AP2A1, B4GALT1, CFP, OSM, and PRIC285. One further gene was significantly down-regulated only in FIGO III/IV EOC patients, NOXA1. In addition, two genes were significantly up-regulated in FIGO III/IV EOC patients but not in FIGO I/II EOC patients, namely CCR2 and DIS3.

**Table 3:** **Gene names and functions of the 13 genes with mean log**_**2 **_**expression fold changes (A) and six proteins with mean log**_**2 **_**abundance values in controls, FIGO I/II patients, and FIGO III/IV patients (B)**

**A)**							
**ProbeID**	**Gene symbol**	**Gene name**	**Function**	**FIGO I/II**^**a**^	**FIGO III/IV**	**JAK STAT**	**Inflammatory response**
115368	AP2A1	adaptor-related protein complex 2, alpha 1 subunit	Clathrin coat assembly	Down. FC^b^: -0.75	Down FC: -0.82		
142487	B4GALT1	UDP-Gal:betaGlcNAc beta 1,4- galactosyltransferase, polypept. 1	Galactosyltransferase	Down (FC: -0.81)	Down (FC: -0.59)		+
157342	C1orf63	chromosome 1 ORF 63	Unknown	n.s. FC: +0.003	n.s. FC: +0.19		
110071	CCR2	chemokine (C-C motif) receptor 2	Chemokine receptor	n.s. FC: +0.72	Up FC: +0.96	+	+
119290	CFP	complement factor properdin	Alternative pathway for complement activation	Down FC: -1.06	Down FC: -1.03		+
105743	DIS3	DIS3 mitotic control homolog (*S. cerevisiae*)	RNase, part of the exosome complex	n.s. (FC: +0.01)	Up (FC: +0.27)		
228089	NEAT1	non-protein coding RNA 84	Non-coding RNA	n.s. FC: -0.01	n.s. FC: +0.26		
182018	NOXA1	NADPH oxidase activator 1	Activates NADPH oxidases	n.s. FC: -0.52	Down FC: -0.60		
205406	OSM	oncostatin M	IL-6 family cytokine	Down FC: -2.62	Down FC: -2.65	+	
161567	PAPOLG	poly(A) polymerase gamma	Poly(A) polymerase	n.s. FC: -0.28	n.s. FC: -0.34		
162222	PRIC285	peroxisomal proliferator-activated receptor A interacting complex 285	Nuclear transcriptional coactivator for several nuclear receptors	Down FC: -2.24	Down FC: -2.33		
109227	ZNF419	zinc finger protein 419	Zinc finger protein	n.s. (FC: -.19)	n.s. (FC: +0.21)		
713562	BC037918	(no ORF in transcript BC037918)	Non-coding RNA	n.s. FC: +0.06	n.s. FC: +0.52		
**B)**							
**Protein**	**Control**	**FIGO I/II**	**corr. p**^**c**^	**FIGO III/IV**	**corr. p**^**c**^		
log2 MIF	8.86	9.67	0.028	9.25	0.040		
log2 prolactin	4.70	6.26	<0.001	6.79	<0.001		
log2 CA125	3.83	7.24	<0.001	8.52	<0.001		
log2 leptin	3.92	2.78	0.033	2.15	<0.001		
log2 osteopontin	3.84	4.59	0.067	5.08	<0.001		
log2 IGF2	10.94	9.10	<0.001	9.16	<0.001		

^a^Significant down- or up-regulation in blood cells of EOC patients compared to healthy blood donors (*t*-test, corrected for multiple testing; n.s., not significant). ^b^FC are actually log_2_-FC values.

^c^Compared to control values.

**Figure 3:**
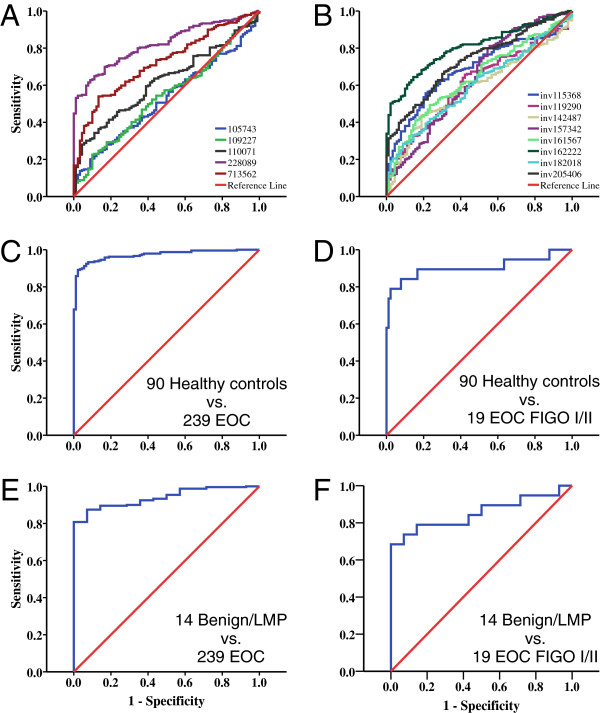
**Classifier performance of single genes and classifier models.** Area under the receiver operating characteristic (ROC) curves (AUCs) for (**A**) the five positive predictive genes, (**B**) the eight negative – thus inverted – predictive genes, (**C-F**) the LASSO estimated risk score built from the 13 blood based expression values used (C) for differentiation of healthy controls and patients with malignant disease, (**D**) for differentiation of healthy controls and FIGO I + II patients, (**E**) for differentiation of patients with benign or low malignant potential tumors and patients with malignant tumors, and (**F**) for differentiation of patients with benign or low malignant potential tumors and FIGO I + II patients.

The expression of five genes was associated with higher probability of EOC (Figure 3A), two of them non-significantly (DIS3 and ZNF419), and eight genes were negatively correlated with the probability of EOC. Using L1 penalized logistic regression, a predictive model was built to discriminate between healthy blood donors as controls and the 239 EOC patients. The model selected all 13 genes including the genes which were not significantly different in the univariate analyses (Table 2). CFP was the only gene whose predictive value changed from its negative direction in the univariate analysis to a positive contribution in the L1 penalized multivariable logistic model.

Since the healthy donors were significantly younger than the EOC patients (Table 1), we investigated whether the risk score from the L1 penalized logistic regression model (i. e., the sum of each subject's gene expressions weighted by the L1 model coefficients) was correlated to age. This was not the case, as confirmed by irrelevant correlation coefficients of the risk score with age of 0.083 (p = 0.449) in healthy donors and 0.104 (p = 0.111) in EOC patients, which indicates clearly the independence of our models from the impact of age on diagnosis of EOC.

The same model discriminated FIGO I + II patients from controls with a sensitivity of 74% at a specificity set at 99% (Figure 3D, AUC = 0.905, CI_95%_ 0.781–1.000, Table 4). However, our model could not discriminate well between healthy controls and patients with benign or LMP tumors (AUC = 0.658, p = 0.058). Nevertheless, malignant tumors were distinguished from benign or LMP tumors with a sensitivity of 87% at a specificity fixed at 95% (AUC = 0.939, CI_95%_ 0.902–0.976) (Figure 3E, Table 4) and even FIGO I + II EOC tumors were different from benign or LMP tumors with an AUC of 0.853 (CI_95%_ 0.719–0.987) (Figure 3F, Table 4). Substantial differences for histological types or grades for all tumors and FIGO I + II stage tumors were not obvious, taking into account the small number of observations in some groups.

**Table 4:** Area under the receiver operating characteristic curves (AUC) of the 13 single genes and the L1 model of these genes

**ProbeID (90 Healthy vs. 239 EOC)**	**AUC**	**Asymptotic Sig. [p-value]**	**Asymptotic 95% confidence interval**	
			**Lower bound**	**Upper bound**
105743	0.525	0.484	0.460	0.590
109227	0.541	0.249	0.475	0.608
110071	0.618	0.001	0.556	0.680
228089	0.822	<0.001	0.778	0.866
713562	0.721	<0.001	0.665	0.778
inv115368	0.684	<0.001	0.625	0.744
inv119290	0.610	0.002	0.546	0.674
inv142487	0.589	0.013	0.525	0.653
inv157342	0.638	<0.001	0.568	0.707
inv161567	0.639	<0.001	0.576	0.702
inv162222	0.804	<0.001	0.758	0.851
inv182018	0.600	0.005	0.537	0.664
inv205406	0.731	<0.001	0.675	0.786
L1 model (LASSO penalty)				
Healthy vs. EOC	0.971	<0.001	0.956	0.987
Healthy vs. FIGO I + II	0.905	<0.001	0.781	1.000
Benign/LMP vs. EOC	0.939	<0.001	0.902	0.976
Benign/LMP vs. FIGO I + II	0.853	0.001	0.719	0.987

### Combination with plasma protein abundance-based biomarkers

**Figure 4:**
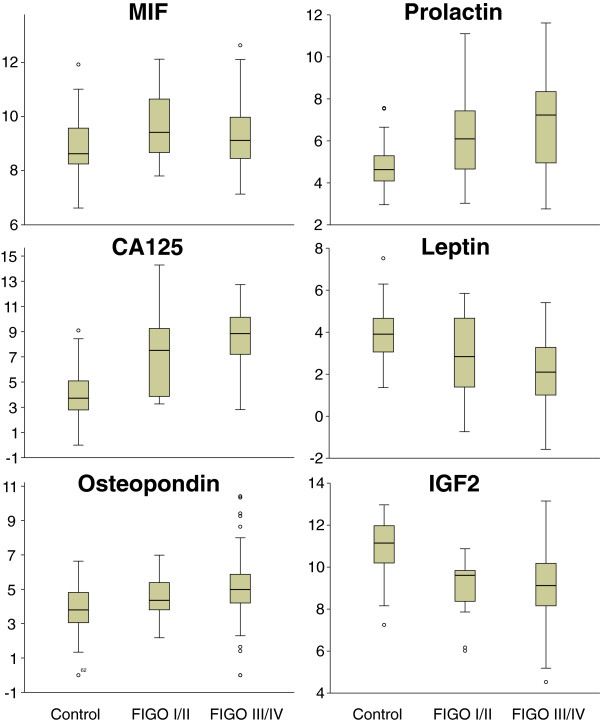
**Boxplots of log**_**2 **_**plasma abundance values for proteins, MIF, prolactin, CA125, leptin, osteopondin, and IGF2 in plasma of controls, and FIGO I/II and FIGO III/IV patients.**

To combine the information of the 13 expression based biomarkers with plasma protein biomarkers, the abundances of six proteins from a known cancer biomarker panel were determined from 224 EOC-plasma samples and from 65 controls (cohort 2) using a commercially available Luminex-based multiplex assay (Figures 2 and 4). In Table 5 the coefficients of the L1 and L2 penalized models, in Figure 2 the corresponding AUC-values, and in Figure 1 the ROC-curves are shown. In Table 6 the characteristics of the two regression models (L1 and the L2 penalized)–are tabularized using the combination of both types of biomarkers. The discriminatory models built from the 13 expression based biomarkers combined with the plasma protein biomarkers proved to be significantly better than the models built from the plasma protein biomarkers alone (p < 0.0001, likelihood ratio test).

**Table 5:** Coefficients of all diagnostic models using either only expression values, protein abundance values, or both types of values in combination (65 controls vs. 224 EOC samples)

**Genes/Proteins**	**Coefficients (L1 and L2 penalized regression)**					
	**L1 7 Genes**	**L2 13 Genes**	**L1 4 Proteins**	**L2 6 Proteins**	**L1 5 Genes 5 Proteins**	**L2 13 Genes 6 Proteins**
105743	0.02	0.63				0.26
109227		0.05				−0.01
110071	0.17	0.26				0.16
228089	0.91	0.99			0.63	0.70
713562	0.34	0.34			0.22	0.29
115368		−0.18				0.36
119290		0.28				0.34
142487		0.15				0.12
157342	0.36	0.64			0.35	0.55
161567		−0.56				−0.27
162222	−1.34	−1.34			−1.58	−1.30
182018		−0.38				−0.09
205406	−0.83	−1.05			−0.54	−0.87
log_2_ MIF				0.09	−0.05	−0.24
log_2_ prolactin			0.67	0.62	0.47	0.53
log_2_ CA125			0.71	0.67	0.33	0.39
log_2_ leptin			−0.32	−0.35	−0.55	−0.59
log_2_ osteopondin				0.05		−0.02
log_2_ IGF2			−0.31	−0.35	−0.47	−0.51
Intercept	3.93	4.90	−2.71	−2.77	4.91	7.31

**Table 6:** Characteristics of both combined models for diagnosis of EOC

**Model**	**L1 lasso penalty**	**L2 ridge penalty**
Blood expression values	5 genes	13 genes
Plasma protein values	5 proteins	6 proteins
AUC^a^ (FIGO I-IV)	0.998	0.998
AUC (FIGO I-II)	0.976	0.979
Specificity (set)	99.6%	99.6%
Sensitivity	97.8%	95.6%
Classification error (bootstrap .632+)	3.1%	2.8%

^a^Bootstrap .632+ validated area under the ROC curve.

### Bootstrap validation

The ability of the two combined models to discriminate cancer patients from healthy controls (ROC analysis), and their classification errors were estimated using bootstrap .632+ validation, simulating external validation by resampling. This corrects for the over optimism that would result from an internal validation of our results (Table 6).

The L1 model, comprised of five gene expression and five protein abundance based values (excluding osteopontin), proved to be slightly more sensitive (97.8% compared to 95.6% at a given specificity of 99.6%). The L2 model, using all 13 gene expression and all six protein abundance values, resulted in less misclassification (bootstrap .632+ cross-validated classification error of 2.8% vs. 3.1%).

## Discussion

In this study, the combination of gene expression values with a serum protein biomarker panel significantly increased the capacity to distinguish between EOC patients and controls.

Serum proteins used for serum based tests are thought to be derived from the tumor-microenvironment and are therefore directly correlated with the amount of tumor mass. We speculate that among others, differences in leukocytes expressions, representing the systemic status of the immune system, are also driven by the malignant processes. Therefore, discrimination between benign and malignant tumors could probably be easier using leukocyte expression patterns than with only serum protein patterns, especially to detect patients with early EOC stages.

Applying a whole genome transcriptomics approach, we identified gene expression patterns of 7 or 13 genes in a leukocytes fraction from peripheral blood, discriminating healthy controls and patients with benign diseases from EOC patients. We are the first to show that this discrimination can be performed with an AUC of 0.984 (CI_95%_ 0.972–0.996) and 0.987 (CI_95%_ 0.976–0.997), respectively. We reached a sensitivity of 88% at a specificity set at 99%. A limitation of this study is that our models were not tested with other cancer entities and thus not enough evidence for cancer-type specificity can be provided. Furthermore, patients with other diseases, i.e. diseases which are inflammatory active like arthritis, should be included in a further – larger – control cohort. Therefore, the term 'specificity' is only related to the statistical differentiation between the controls and the ovarian cancer patients of this study. The diagnostic power of this gene expression patterns is similar or even stronger to marker panels derived from serum proteins [7,10-12]. Furthermore, our gene expression model can distinguish benign or LMP tumors from malignant tumors with a rather high sensitivity and specificity (87% and 95%, respectively). Only Zhang at al. [7] had tested their multi-marker serum panel for the discriminatory potential between benign or low malignant potential tumors and malignant tumors, with sensitivities and specificities in the range of 33% to 50% and 33% to 45%, respectively.

Combining the expressions of the 13 genes that we have identified with the protein abundance values from a commercially available serum protein biomarker panel significantly increases our predictive model. A model comprised of five gene expression values and five protein abundance values showed a sensitivity of 97.8 at a specificity of 99.6%. The high sensitivity and specificity reached by our models highlight a possible applicability of our combined model as a diagnostic test in high-risk individuals or as second test in combination with a CA-125/transvaginal sonography-based screening approach. The bootstrap .632+ validated classification error for this model was 3.1%. Our models were not tested with other cancer entities and thus our study does not provide enough evidence for cancer-type specificity. Hence, our use of the term 'specificity' relates to the statistical differentiation between the controls and the ovarian cancer patients of this study only. Nevertheless, we think that the combination of a sensitive blood gene expression test (even if it is not cancer type – or even – cancer specific) with a cancer type specific protein test provides in combination both, a high sensitivity and a high specificity.

The functional specifications of the 13 genes that we identified are widespread among the pool of functional clusters and pathways, which is not a big surprise given the model building approaches used for generating the discriminative models, i.e. methods which exclude correlated genes from the final model explicitly (Table 3). Nevertheless, four genes are involved directly in inflammatory response and the immune system (B4GALT1, CCR2, CFP, and OSM), and two of them in the JAK/STAT pathway (CCR2 and OSM), known to be a common signaling pathway used by many cytokines [21]. Two genes seem to be non-coding RNAs (NEAT1 and transcript BC037918), presumably involved in regulation of transcription. The other protein functions are completely incoherent: one is a zinc-finger protein (ZNF419) of unknown function, one a poly(A) polymerase (PAPOLG), one a co-activator for several nuclear receptors like PPARA, PPARG, TR-beta-1, ER-alpha, and RXR-alpha (PRIC285), one a activator of catabolic NADPH oxidases (NOXA1), one is an RNase enzyme and can be part of the exosome complex (DIS3), and one is involved in the assembly of clathrin coated vesicles (AP2A1). From one transcript, C1orf63, no homologue protein is known.

The next step will be to validate the combined gene expression- and protein abundance- based predictive model using an independent large cohort of various controls and patients including patients with a systematic inflammatory status and including a larger sample of patients with FIGO I/II stages, which is an apparent shortcoming of this study.

## Conclusion

The combination of two different types of biomarker signatures, one derived from blood plasma and one derived from the peripheral immune system, improved the discriminative power between control persons and ovarian cancer patients significantly, compared to the two single signatures alone. The concept of combining different types of biomarker (signatures) for one diagnostic or prognostic test opens new avenues, particularly by expanding this concept to further types of blood based biomarker, e.g. derived from circulating tumor cells or cell-free nucleic acids and involving genetic, epigenetic, or microRNA associated biomarker (signatures).

## Abbreviations

EOC: Epithelial ovarian cancer; RT-qPCR: Reverse transcriptase quantitative polymerase chain reaction; OS: Overall survival; SELDI: Surface-enhanced laser desorption/ionization; RCA: Rolling circle amplification; LMP: Low malignant potential, OVCAD, OVarian CAncer: Diagnosis of a silent killer; EDTA: Ethylenediaminetetraacetic acid; USC: Uncorrelated shrunken centroids; ANOVA: Analysis of variance; SAM: Significance analysis of microarrays; ROC: Receiver operating characteristic; AUC: Area under the ROC curve; CI: Confidence interval; FIGO: International Federation of Gynecology and Obstetrics; FC: Fold change.

## Competing interests

A patent was filed (DP, DT, RZ).

## Authors’ contributions

DP, DT, and RZ concepted and designed the study. DP, GH, ESch, and AW performed microarray and RT-qPCR experiments. EO performed the multiplexed immunoassays. DP made the microarray bioinformatics and DP, GH, and MK analyzed the data statistically. SA, JS, IB, IV, TVG, SM, NC, and PS recruited patients, collected patient samples and data, and revised the manuscript critically. DP, SA and RZ wrote the manuscript and all authors read and approved the final manuscript.

## Pre-publication history

The pre-publication history for this paper can be accessed here:

http://www.biomedcentral.com/1471-2407/13/178/prepub
